# Pathological predictors for curettage and cementation outcome in proximal tibial giant cell tumors

**DOI:** 10.6026/973206300210190

**Published:** 2025-02-28

**Authors:** Deepak Kumar, Ashish Kumar, Nilam Bhasker, Sanjeev Kumar, Atin Singhai, Priyank Pratap, Ankit Sriwastava, Madhusudan Mishra

**Affiliations:** 1 Department of Orthopaedic Surgery, King George's Medical University, Lucknow, Uttar Pradesh-226003, India; 2 ESIC Hospital, ESIC Hospital Complex, Sarojini Nagar, Lucknow, Uttar Pradesh-226023, India; 3 Department of Pathology, King George's Medical University, Lucknow, Uttar Pradesh-226003, India

**Keywords:** Giant cell tumor (GCT), tumor grading, recurrence risk, cortical bone involvement pathological predictors, functional outcomes

## Abstract

Proximal tibia giant cell tumors (GCT) are aggressive with high-recurrence rate, function-affecting and benign neoplasms. Therefore, it is of interest to report the pathological predictors for curettage and cementation outcome in proximal tibial giant cell tumors. Hence, 32 patients treated with curettage, poly-methyl-methacrylate (PMMA) cementation and locking plate fixation between 2018 and 2022 was included in this study. The average age of patients was 28.1 ± 6.9 years and most tumors were Grade 2 (campanacci grade) with 62.5% cortical involvement. The average musculo-skeletal tumor society (MSTS) score was 27.2 ± 4.2 with acceptable function. Thus, tumor grade and cortical involvement were the main predictors of recurrence, reflecting the need for targeted treatment.

## Background:

Giant cell tumors of bone are rare, benign but locally aggressive neoplasms. They occur mainly in the epiphyseal region of long bones in young adults [1]. The most frequently affected site is the proximal tibia because of its load-bearing nature and the intricacies of its biomechanics [2]. Giant cell tumors are difficult for the clinician because they are aggressive, have a tendency for local recurrence and may cause significant functional morbidity. Although benign, about 3% of giant cell tumors metastasizes to the lungs, which have complicated their management [3]. The treatment of giant cell tumor has evolved whereby curettage and cementation have emerged as the preferred methods while preserving joint function when thorough excision is not obligatory [4]. Curettage involves surgical removal of the Tumor where possible without causing much damage to the surrounding bone and other soft tissues. Apart from enhancing local control, adjuvants such as PMMA cement provide structural support to the defect created after curettage [5]. Cementation provides immediate stability, which makes it possible to achieve early weight-bearing significant benefits in load-bearing bones like the proximal tibia [6]. However, the major defects caused by curettage compromise the mechanical stability and thus other measures such as plate fixation are required to strengthen the compromised bone [7]. Pathological predictors including Tumor size, cortical breach and soft tissue extension are significant considerations that would affect the prognosis of surgery [8]. These parameters affect not only the degree of resection but also the recurrence and functional outcome. Though many studies were conducted on the efficacy of cement augmentation alone, scant data are documented about curettage coupled with cementation combined with plate fixation in proximal tibial giant cell tumors. Understand plate application, particularly in cases where there has been complete bone loss or weakened structures, as the application would serve to avoid postoperative fractures and thus enhance better recovery of functionality [9]. Therefore, it is of interest to evaluate the pathological predictors and clinical outcomes associated with curettage, cementation and plate application in managing giant cell tumors of the proximal tibia.

## Materials and Methods:

It is a retrospective cohort study, conducted on 32 patients diagnosed with giant cell tumor s of the proximal tibia, managed between January 2018 and December 2022 at a single tertiary care institution. This study was approved by the institutional ethics review board and informed consent was obtained from all the patients before the surgery. The criteria for inclusion were patients who had histologically confirmed giant cell tumor s and underwent an extended curettage procedure, bone grafting, cementation and internal fixation using a locking compression plate. Excluded were those with secondary malignant transformation, distant metastases at presentation, or incomplete follow-up data.

## Surgical technique:

All the surgeries were done under spinal anaesthesia by a standard anterolateral approach to the proximal tibia. Extended curettage was done by extending the walls of the cavity to the longest dimension of the lesion followed by thorough irrigation with pulsatile lavage to remove the residual tumor tissue. Gel foam was applied to areas of the cortical breach and the subchondral region to support haemostasis. Subchondral bone grafting was done with subsequent cementation using PMMA for the restoration of the structure. Curettage was carried out with high-speed burs operating at 75,000-80,000 RPM for the complete removal of tumor tissue. Locking compression plates were used for internal fixation to increase mechanical stability, especially in cortical bone compromise cases.

## Adjuvant therapy:

All patients were given adjuvant zoledronic acid. The preoperative regimen contained 5 mg intravenous infusions, which were administered weekly for three weeks. Postoperatively, zoledronic acid was administered every third month for one year; this treatment was aimed at reducing the risk of tumor recurrence and supporting bone healing.

## Data collection:

Demographic data, clinical characteristics and operative details were obtained from the medical records. The grading of the Tumor was based on the Cam Panacci classification system. Intraoperative data included the duration of surgery and the estimated blood loss. Postoperative follow-up data included functional outcomes evaluated by the musculoskeletal tumor society scoring system, complications and recurrence rates. Follow-up ranged from 2 to 6 years.

## Outcome measures:

Primary outcomes included local recurrence rates and functional outcomes as measured by musculoskeletal tumor society scores. Secondary outcomes included intraoperative and postoperative complications, such as infection and shaft Tissue Recurrence. Descriptive statistics were used for summarizing demographic data as well as clinical outcomes. Continuous variables were reported with mean values and standard deviation, whereas categorical variables were reported as percentages. A statistical package SPSS, version 25.0 software was used and at a significance level of p < 0.05.

## Results:

**Figure 1:**
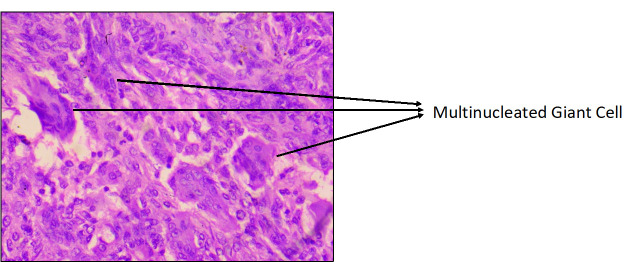
Clinico-radiological outcome of a grade 2 giant cell tumor of the proximal tibia. Preoperative x ray of the knee joint of 35-year male (a), showing lytic lesion of proximal tibia suggestive of giant cell tumors (grade 2) confirmed by histopathological examination (b), managed with extended Curettage, Autogenous Bone Grafting, Cementation and Internal Fixation application, (c) Post-op X-Ray (d) 2-year follow-up (e) Functional outcome at 2 Year

**Figure 2:**
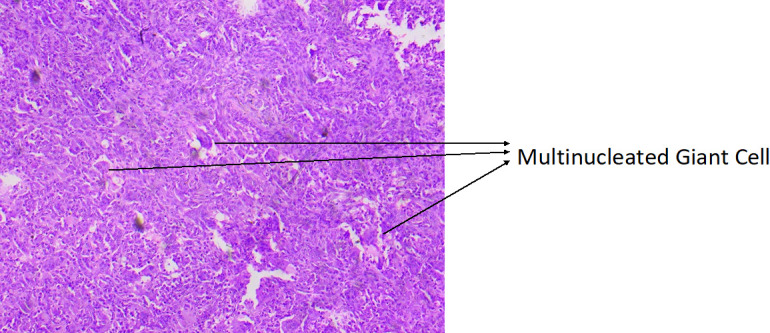
Clinico-radiological outcome of a grade 2 giant cell tumor of the proximal tibia. Preoperative x-ray of the knee joint of a 27-year female (a), showing lytic lesion of proximal tibia suggestive of giant cell tumors (Grade 2) confirmed by histopathological examination(b), managed with extended Curettage, Cementation and plate application, (c) 2-year follow-up, (d) 5-year follow-up, (e, f) Functional outcome at 5 years

A total of 32 patients with a mean age of 28.12 ± 6.94 years and a range of 18-42 years, participated in the study. Gender-wise distribution showed that males constituted 56.25% (n=18) while females accounted for 43.75% (n=14). The study revealed that giant cell tumors of the proximal tibia were more common on the right side, that is, 56.25% (n=18), whereas the left side accounted for 43.75% (n=14). Tumor grading by Campanacci grading showed Grade 1 in 12.5% (n=4), Grade 2 in 75% (n=24) and Grade 3 in 12.5% (n=4). Cortical bone breach/involvement was found in 62.5% (n=20) and no cortical involvement was present in 37.5% (n=12) of cases, respectively (Table 1). The procedures involved included Extended curettage, bone grafting and bone cementing with internal fixation using a locking compression plate in 68.75% (n=22) of the cases (Figure 1) and extended curettage with bone cementing and internal fixation using a locking compression plate in 31.25% (n=10) (Figure 2). Subchondral bone grafting was carried out in cases where, after curettage, the subchondral bone thickness was less than 0.8 cm or 8 mm. The same criterion was applied to all patients with tumors graded Grade 2 and Grade 3 in the Campanacci grading system. The procedure of subchondral bone grafting was not carried out in 12.5% (n=4) of Grade 1 and 18.75% (n=6) of Grade 2 cases. All the patients were given adjuvant Injection Zoledronic Acid 5 mg, 100% (n=32). The mean duration of surgery was 1.30 ± 0.26 hours with an average blood loss of 154.37 ± 8.00 ml with a range of 150-170 ml. The mean Musculoskeletal Tumor Society score was 27.18 ± 4.22 with a range of 15-30. The average follow-up period was 3.93 ± 1.38 years with a range of 2-6 years (Table 1). Complications included soft tissue recurrence in 6.25% (n=2) of cases and infection in 6.25% (n=2) of cases (Table 2).

## Discussion:

The treatment of giant cell tumor in the proximal tibia remains one of the problems, largely due to the tendency towards locally aggressive behaviour of this tumor and its associated risks of functional impairment and recurrence. The study intended to investigate the efficacy of a combined curettage, cementation and plate fixation technique by identifying pathological predictors as well as clinical outcomes. Pathological predictors that can predict recurrence in giant cell tumors include tumor grade, cortical breach and soft tissue extension. Elevated tumor grades, especially Grade 3 as categorized by Campanacci's classification, demonstrate an increased level of local aggressiveness and an augmented likelihood of recurrence [2]. The involvement of cortical bone serves as a crucial indicator of recurrence, as it offers a route for the survival and dissemination of tumors [10]. In the current study, recurrence was evident only in Grade 3 tumors with cortical breach, further reinforcing the earlier literature, where the higher-grade tumors have shown more aggressive features. Moreover, soft tissue invasion and cortical destruction have a poor prognosis with decreased local control and increased rates of recurrence [11]. These observations critically reflect the need for appropriate characterization of the tumors preoperatively to ensure directed intervention that avoids recurrence.

The postoperative recurrence rate was 6.25%. Adjuvant zoledronic acid (5 mg intravenous slow infusion weekly for three weeks) was given in these cases to aid bone healing and reduce recurrence by inhibiting osteoclast activity, as done by van der Heijden *et al.* [12] and Pannu [13]. Recurrences were addressed by the second surgery which involved removal of the soft tissue mass that helped in eliminating the remaining tumor cells and improved local control. Infection was one of the complications that developed in 6.25% of the cases. Based on culture sensitivity reports, appropriate antibiotic therapy was administered. In one case of scar dehiscence, infected cement and plate were removed. The procedure that followed was re-curettage, new cementation and plate fixation. This method was important in restoring mechanical stability and reducing the chances of further infection since under-excision of infected tissue might worsen complications. Curettage with cementation and plating has been a well-recommended method for proximal tibial giant cell tumors. This approach does provide structural stability, hence reducing the risk of recurrence. Campanacci *et al.* [2] and Saibaba *et al.* [14] have proved the efficacy of wide curettage with adjuvant therapy such as cementation and internal fixation for local control. The current study highlighted the fact that cortical involvement requires the supplementation of plate fixation, especially in cases where there is compromise of the cortex. The mean MSTS score of 27.18 ± 4.22 showed excellent functional outcomes, which are like those reported by Zhou *et al.* [15]. Despite the fear of developing osteoarthritis following subchondral cementation, our mid-term follow-up demonstrated minimal degenerative changes. Wechsler *et al.* [16] established potential risks for cementation-related articular degeneration. However, those risks were not present in relatively young cohort of patients.

## Conclusion:

Curettage, cementation and plate fixation provide favorable functional outcomes and low recurrence rates in proximal tibial giant cell tumors. Hence, pathological predictors like tumor grade and cortical breach require targeted interventions. Thus, proper management ensures long-term stability and better patient outcomes.

## Figures and Tables

**Table 1:** Demographic detail of the subjects

**Characteristics**	**Value**	**Statistics**
Age Mean		28.12 ± 6.94 (Range 18-42)
Gender	Male (n=18)	56.25%
	Female (n=14)	43.75%
Diagnosis (Giant cell tumour of proximal tibia)	Left (n=14)	43.75%
	Right (n=18)	56.25%
Tumour grade campanacci grading 1, 2 and 3	Grade 1 (n=4)	12.50%
	Grade 2 (n=24)	75%
	Grade 3 (n=4)	12.50%
Cortical bone involvement / Breech	Involved (n=20)	62.50%
	Not Involved (n=12)	37.50%
	Extended curettage, bone grafting and bone cementing	68.75%
	with internal fixation by locking compression plate (n=22)	
**Procedure**		
	Extended curettage and bone cementing with	31.25%
	internal fixation by locking compression plate (n=10)	
Without Subchondral Bone Grafting	Grade 1 (n=4)	12.50%
	Grade 2 (n=6)	18.75%
Adjuvant therapy	Inj. Zoledronic 5 mg (n=32)	100%
Duration of surgery (hours)		1.30 ± 0.26 (1-1.8 Hours)
Blood loss (ml)		154.37 ± 8.00 (150 ± 170 ML)
MSTS score		27.18 ± 4.22 (Range 15-30)
Follow-up (in Year)		3.93 ± 1.38 (Range 2-6 Years)

**Table 2:** Complications associated with surgical management

**Characteristics**	**Severity Grade**	**Value (n)**	**Statistics**
Soft Tissue Recurrence	3	2	6.25%
Infection	2	2	6.25%
